# Fragility Fractures of the Pelvis With Acetabular and Femoral Neck Fractures in Elderly Patients

**DOI:** 10.7759/cureus.86065

**Published:** 2025-06-15

**Authors:** Takuya Nakai, Shigeo Fukunishi

**Affiliations:** 1 Orthopedic Surgery, Nishinomiya Kaisei Hospital, Nishinomiya, JPN

**Keywords:** acetabular fracture, femoral neck fracture, fragility fractures, pelvic fracture, total hip arthroplasty

## Abstract

Fragility fractures of the pelvis (FFP) in the elderly have been on the rise. Conventional treatment options have often been conservative. However, in recent years, osteosynthesis has been recommended in order to achieve early ambulation.

Surgical treatment was performed for combined FFP, femoral neck and acetabular fractures in elderly patients who did not have a clear history of trauma. In this case report, we describe two cases of combined fractures of FFP.

Patient 1 was diagnosed with a combined fracture of the femoral neck and acetabulum, classified according to Rommens classification as III-c, and following osteosynthesis for a posterior column fracture, underwent allogenic bone grafting and total hip arthroplasty (THA) with a Burch-Schneider cage.

Patient 2 was diagnosed with a combined fracture of the femoral neck and acetabulum, classified according to Rommens classification as II-b. The patient underwent THA with an allogenic bone graft and a Kerboull-type plate.

Surgical treatment with THA using an acetabular reinforcement device has been successful in rare cases with combined fractures of the FFP, femoral neck and acetabulum.

## Introduction

Fragility fractures of the pelvis (FFP) in the elderly have been on the rise in recent years [1-4]. Conventional treatment options have often been conservative, such as bed rest and subsequent rehabilitation. However, conservative treatment leads to increased length of hospital stay, decreased activities of daily living (ADL), and increased mortality, which may increase the burden on the medical and social security systems [4-6]. Rommens et al. proposed a classification of FFP fracture types with surgical indications, which has been widely cited [7]. In recent years, there have been increasing reports of osteosynthesis as a treatment option for FFP due to early rehabilitation and early return to society [8-10]. On the other hand, acetabular fractures in the elderly are also increasing and surgical treatment of these fractures with osteosynthesis or combined osteosynthesis and total hip arthroplasty (THA) has been reported to produce satisfactory results [11-13]. Fractures of the combined acetabular and pelvic ring are commonly caused by high-energy trauma, such as traffic accidents and falls from a significant height. In this case report, we describe two cases of combined fractures of FFP, acetabular and femoral neck fractures in elderly patients who underwent surgical treatment despite having no clear history of trauma.

## Case presentation

Case 1

**Figure 1:**
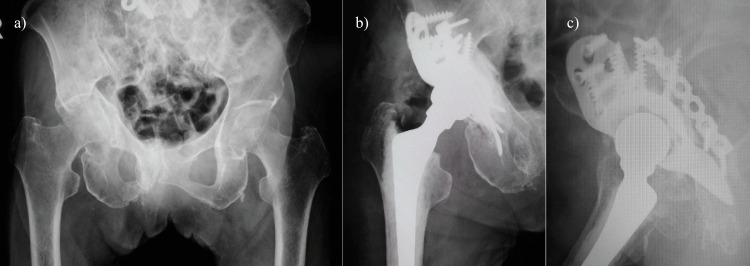
Radiographs of case 1: a 78-year-old female patient a) Preoperative radiograph, b) Postoperative radiograph (anterior-posterior view), and c) Postoperative radiograph (lateral view)

**Figure 2:**
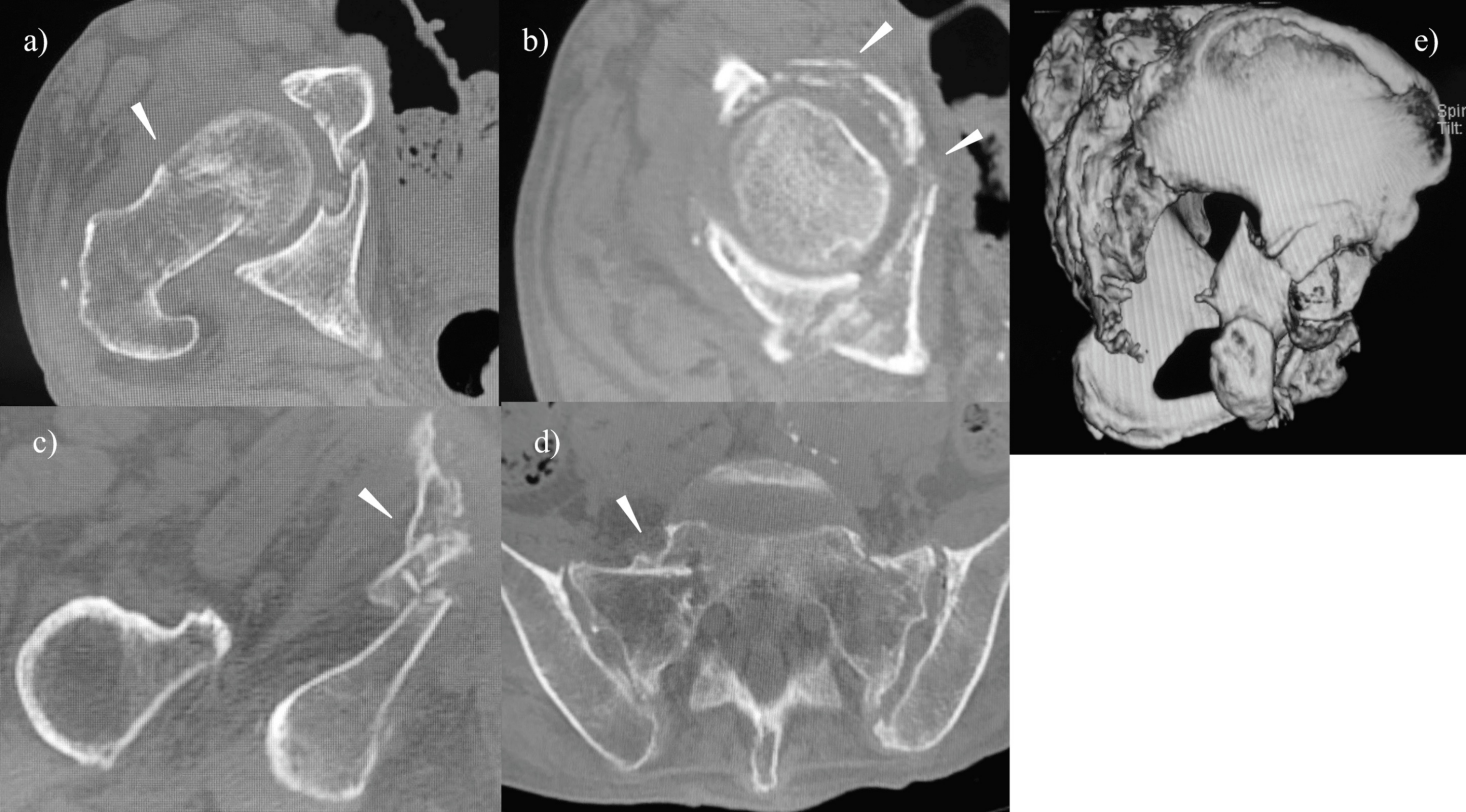
Preoperative CT of case 1 The white arrow indicates a) femoral neck fracture, b) acetabular fracture, c) pubic ramus fracture, and d) sacral ala fracture, and e) a 3D CT scan posterior column fracture was identified

Case 1 was a 78-year-old woman diagnosed with rheumatoid arthritis. The patient had been taking 8 mg of methotrexate a week and 2 mg of prednisone a day for rheumatoid arthritis since she was 65 years old. There was no history of falls or other minor trauma. Three weeks before visiting, the patient noticed pain in her right hip joint, and the pain gradually became more severe, making it difficult to walk. A plain radiograph and a CT image of the hip showed fractures in the acetabulum, femoral head and neck, sacrum, and the pubic ramus, which was classified as FFP III-c according to the Rommens classification system (Figures 1a, 2).

Bone mineral density at the time of admission was 0.725 g/cm^2^ in the spine and 0.515 g/cm^2^ in the contralateral femoral neck. Prior to the visit to our hospital, the patient had not received any treatment for osteoporosis. THA combined with osteosynthesis was selected as the surgical option. After osteosynthesis of the posterior column fracture, allogeneic bone grafting and using the Burch-Schneider cage (Zimmer Biomet, Warsaw, IN, USA) was performed for reconstruction of the acetabulum. Cemented THA with collarless polished tapered stems, polyethylene liner, and metal head (Zimmer Biomet, Warsaw, Indiana) was performed (Figures 1b, 1c). The operating time was 122 minutes, intraoperative blood loss was 496 ml, and 400 ml of blood was transfused during surgery. Treatment for osteoporosis with teriparatide acetate and vitamin D was started after surgery. The patient was permitted to start gait training two weeks after surgery and advanced to full-weight bearing one month after surgery. The patient's ADL achieved walker ambulation at four years postoperatively until her death at age 82. The modified Harris hip score (HHS) was 55 points at three years after surgery and at the time of the final follow-up.

Case 2

**Figure 3:**
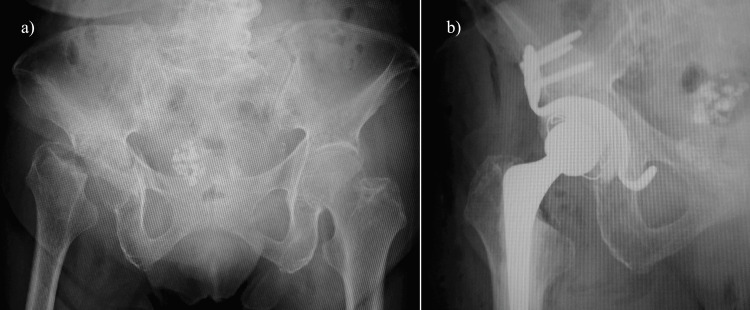
Radiographs of case 2: an 82-year-old female patient a) Preoperative radiograph and b) Postoperative radiograph

**Figure 4:**
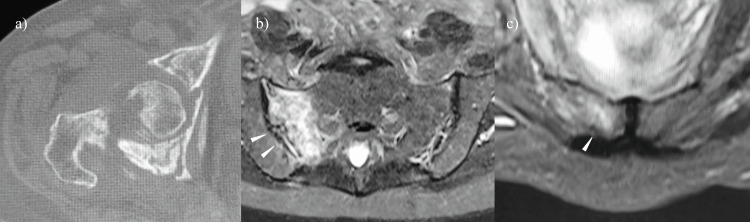
Preoperative CT and MRI of case 2 a) Preoperative CT and b, c) MRI of the T2-weighted STIR sequence The white arrow indicates b) sacral ala fracture and c) pubic ramus fracture STIR: Short Tau Inversion Recovery

Case 2 was an 82-year-old woman. The patient was admitted to the department of internal medicine for the purpose of detailed examination due to hemorrhage. One month prior to admission, the patient had no history of falling but was aware of pain in the right hip joint and was diagnosed with lumbar stenosis, for which NSAIDs were prescribed. The patient could walk with a walker prior to hospitalization. A CT scan of her abdomen to examine for gastrointestinal bleeding revealed a fracture in the hip. The cause of the hemorrhage was bleeding from a colonic diverticulum, and there was no malignancy. The patient was transferred to our hospital for surgical treatment. A plain radiograph and CT images of the right hip revealed fractures in the acetabulum and femoral neck. Although undetected by a plain radiograph and CT, MRI showed fractures in the sacrum and right pubic ramus (Figures 3a, 4), which were classified as FFP II-b according to the Rommens classification system.

Bone mineral density upon admission was 1.063 g/cm^2^ in the spine and 0.572 g/cm^2^ in the contralateral femoral neck. Prior to transfer to our hospital, the patient had not received any treatment for osteoporosis. Allogeneic bone graft and Kerboull-type plate (Kyocera Medical, Osaka, Japan) were used for acetabular reconstruction. In addition, cemented THA was performed with a rim fit cup, Exeter stem, and ceramic head (Stryker Orthopedics, MI, USA) (Figure 3b). The operative time was 122 minutes, and intraoperative blood loss was 496 ml without transfusion. Treatment for osteoporosis with teriparatide acetate and vitamin D was started after surgery. The patient was permitted to start gait training three days after surgery. The patient was eventually able to walk unassisted, and her modified HHS score was 82.5 points three years after surgery and at the time of her final follow-up.

## Discussion

Pelvic fractures commonly result from high-energy trauma. However, it has been reported that in patients with osteoporosis, they can occur as a result of minor trauma, leading to a significant decrease in the patient’s ADL. The prevalence of these fractures in the elderly has been increasing in recent years [1-4]. The pelvic ring is a continuous ring-shaped structure consisting of the ilium, sacrum, pubis, and ischium. Fractures that disrupt the ring structure may lead to subsequent fractures at other sites, further increasing instability. Scheyerer et al. reported that 96.8% of pubic fractures were accompanied by sacral fractures as revealed on MRI [14]. Fukunishi et al. reported a case of consecutive bilateral pubic and bilateral iliac fractures in an elderly patient with rheumatoid arthritis [15]. On the other hand, Graul et al. stated that acetabular fractures were associated with FFP in 3% of FFP cases and proposed that a combination of FFP and acetabular fractures was rare [16]. Additionally, Laksmanan et al. stated that a pubic fracture is very rarely associated with a femoral neck fracture, and when a pubic fracture is identified, no additional examinations are required to confirm a femoral neck fracture [17]. There is only one paper available that reports on these fractures occurring at the same time, and Adams et al. reported a case of FFP associated with inter-trochanteric fracture of the femur as a result of minor trauma [18]. As mentioned above, it is considered extremely rare for a minor trauma to cause three combined fractures to occur simultaneously FFP, acetabular fracture and proximal femoral fracture. The two cases presented in this case report are examples of this rare occurrence where the patients suffered combined fractures of FFP, the femoral neck and acetabulum without any prior trauma. In insufficiency fractures in osteoporotic patients, the force leading to a fracture is very low, and the patient's own body weight may be sufficient to produce a fracture [7]. Although it is not possible to specify the mechanism of these combined fractures, the fact that the patient in case 1 had been complaining of hip pain for three weeks before the fractures were diagnosed and the patient in case 2 for a month before, suggests that one of the fragility fractures occurred first and gradually led to a chain of other fractures. Rommens states that in the pelvic ring of elderly people, the strength of the bone is weaker than the strength of the surrounding ligamentous components, and mainly only the bone structures are predominantly destroyed, and therefore, the ligaments on the posterior side of the pelvic ring are intact and the anatomical ring structure is maintained, and fractures develop by moving within these ring structures [19]. It is possible that fractures of the fragile bones were repeated one after another within the unstable ring structure. Regarding treatment options, surgical treatment may affect the patient's general condition, while conservative treatment may lead to increasing displacement of the fracture and a decrease in the patient’s ADL as a result of prolonged bed rest. While early mobilization is expected in osteosynthesis, it has been reported that the conversion rate to THA due to postoperative OA is 11.6%, even when anatomical reduction is achieved after osteosynthesis for acetabulum fractures in elderly patients [20]. In the two cases presented here, the acetabular fracture was combined with a Garden IV femoral neck fracture, and a single-stage THA was selected as the surgical option. In case 1, the anterior and posterior columns of the acetabulum were fractured. Osteosynthesis with plate fixation was performed first in order to compensate for posterior instability. Due to the instability of the anterior column, there was concern that placement of a Kerboull-type plate would result in unstable hook fixation on the obturator foramen. Therefore, the acetabulum was reconstructed using a Burch-Schneider cage instead of a Kerboull-type plate during cemented THA. In case 2, the acetabulum was reconstructed with an allogeneic bone graft and a Kerboull-type plate during cemented THA. MRI showed an occult fracture in the pubic ramus, but intraoperative findings showed good stability of the hook and Kerboull-type plate. In the two cases presented, early ambulation and early rehabilitation intervention were possible, with satisfactory results. However, some challenges remain regarding the indications for surgical treatment in the two cases presented here. Had the surgical indications according to Rommens classification been strictly followed, osteosynthesis of the posterior pelvic ring using trans-iliac sacral screws or other devices would have been necessary, and more active rehabilitation might have been possible [7]. On the other hand, in addition to reports that the decision for surgical intervention for FFP is based on the morphology of the fracture, others state that it is based on residual pain after conservative treatment [8-10]. In the present two cases, at least three weeks had passed since the fracture occurred, but no progression of displacement at the fracture site or low back pain was observed. Therefore, osteosynthesis to the posterior pelvic ring was not performed. However, it is possible that weight bearing may have caused progressive displacement of the fracture site, which is an issue for future investigation.

## Conclusions

Two elderly patients with osteoporosis developed a pelvic ring fracture, a proximal femoral fracture, and an acetabular fracture without a history of trauma. Very rare cases of FFP with combined fractures of the femoral neck and acetabulum were successfully treated by surgery. In patients who are in stable general condition, single-stage acetabular osteosynthesis with THA may be the treatment of choice for these complex fractures in the elderly with the goal of early ambulation.
